# Analysis and Improvement of a Dual-Core Photonic Crystal Fiber Sensor

**DOI:** 10.3390/s18072051

**Published:** 2018-06-27

**Authors:** Pibin Bing, Shichao Huang, Jialei Sui, Hua Wang, Zhiyong Wang

**Affiliations:** Institute of Electric Power, North China University of Water Resources and Electric Power, Zhengzhou 450045, China; huangshichao@stu.ncwu.edu.cn (S.H.); suijialei@stu.ncwu.edu.cn (J.S.); zzuwanghua@163.com (H.W.); wangzhiyong130@163.com (Z.W.)

**Keywords:** dual-core photonic crystal fiber, wavelength sensitivity, high linearity

## Abstract

The characteristics of the dual-core photonic crystal fiber (PCF) sensor are studied using the finite element method (FEM), and the structure is improved according to the numerical simulation results. The results show that whether or not the four large air holes far away from the geometry center of the PCF are filled with analyte has no influence on the wavelength sensitivity of the sensor which means those holes can be replaced by small air holes. The wavelength sensitivity can be tuned by adjusting the sizes of the other large air holes which are as for liquid holes. The dynamic detection range of the refractive index (RI) is from 1.33 to 1.51. In particular, high linearity is obtained in the range of 1.44 to 1.51. The sensitivity is as high as 6021 nm/RIU when the liquid holes are the smallest. When liquid holes are tangential with the envelope of first layer air holes, the wavelength sensitivity is 4028 nm/RIU, and the coefficient of determination (R^2^) is 0.99822 when the RI of the analyte varies from 1.44 to 1.51 which shows that high sensitivity and good linearity are both obtained.

## 1. Introduction

The surface plasma is excited on the surface of the metal–dielectric interface which is exceedingly sensitive to changes in the refractive index (RI) of the analyte. A. Hassani and M. Skorobogatiy proposed that the photonic crystal fiber (PCF) sensor plated gold film can detect minute changes in the RI of the analyte [1]. The polished grapefruit fiber structure coating with silver film [2] or bimetallic [3] can obtain a pretty high wavelength sensitivity. Sensors with D-shaped structures, such as air holes arranged in hexagons [4,5] or rectangules [6], central holes filled with high RI liquid [7] and PCF plated with indium tin oxide at near-infrared wavelength [8,9] have been studied in recent years. The polishing fabrication method of the D-shaped optical fiber has also been reported [10]. Although these sensors have the advantages of high sensitivity and convenient liquid filling, the performance of sensors when the RI is greater than 1.42 is not good. In other structures, sensors with a silver–graphene layer could prevent silver from being oxidized [11,12]. The silver film [13] and gold film [14] coated on the outer layer of the PCF have high sensitivities. Three-holed PCF coated with an auxiliary dielectric layer and gold film makes analyte filling easy [15]. However, all of these sensors are not applicable for the detection of high RI.

Zhou et al. [16] studied the hybrid mechanisms of the PCF and pointed out that the fundamental mode can couple with the surface plasmon polaritons (SPP) mode in a higher RI of the analyte, so that the RI of analyte is detected in the range of 1.25 to 1.45. It was shown that the RI from 1.33 to 1.53 could be detected by the multi-core PCF sensor [17]. When the RI is low, the dispersion curves of the fundamental mode intersect with the plasma mode, and the intersection point is a phase matching point, while the real parts of the two modes have no intersection when the RI is higher due to the avoided crossing effect. However, the imaginary parts of the two modes intersect where energy exchanges between them, and the intersection is called the avoided crossing point. An investigation of the solid-core PCF [18] and the novel D-shaped PCF [19] showed that energy exchange occurs at the avoided crossing point. It can be concluded that when the number of fiber cores is more than one or akin to multi-core, the coupling mode, called loss matching, is different from phase matching in higher RIs, which enlarges the RI detection range. In fact, dual-core PCFs have a large quantity of applications, attracting a lot of researchers’ interest. Yu et al. [20] proposed a PCF mode converter. This kind of mode converter was used to implement wideband mode-division multiplexing of few-mode optical fibers. Wang et al. [21] designed a dual-core PCF with a hexagonal lattice used in the field of biomolecule detection due to its high sensitivity. A polarization beam splitter based on dual-core photonic crystal fiber was investigated as well [22]. Moreover, by adjusting the number of fiber cores and the arrangement of air holes, PCFs cannot only be used as surface plasmon resonance sensors [23,24], but also as splitters [25,26], filters [27,28] and power beam combiner [29]. The flexible design of the PCF structure means that PCFs can satisfy some specific functions. It provides a possibility for detecting high refractive index analyte.

In this paper, a dual-core PCF structure is proposed, and the influence of parameters is studied and then the structure is improved. The numerical simulation results show it has no effect on wavelength sensitivity whether the four large air holes far away from the PCF geometry center are filled with analyte or not. As well, the sizes of those holes have no impact on the sensor. The sizes of the other large air holes which are used for liquid holes has an effect on wavelength sensitivity. The smaller the liquid holes are, the higher the sensitivity is. To make the sensor desirable for applications, adjusting the diameter of liquid holes is essential to reduce the filling difficulty. A solution is that liquid holes are tangent with the envelope of the first layer air holes. The wavelength sensitivity is 4028 nm/RIU and R^2^ is 0.99822 when the RI of analyte varies from 1.44 to 1.51, so that the sensor has both high sensitivity and good linearity.

## 2. Design and Analysis

**Figure 1:**
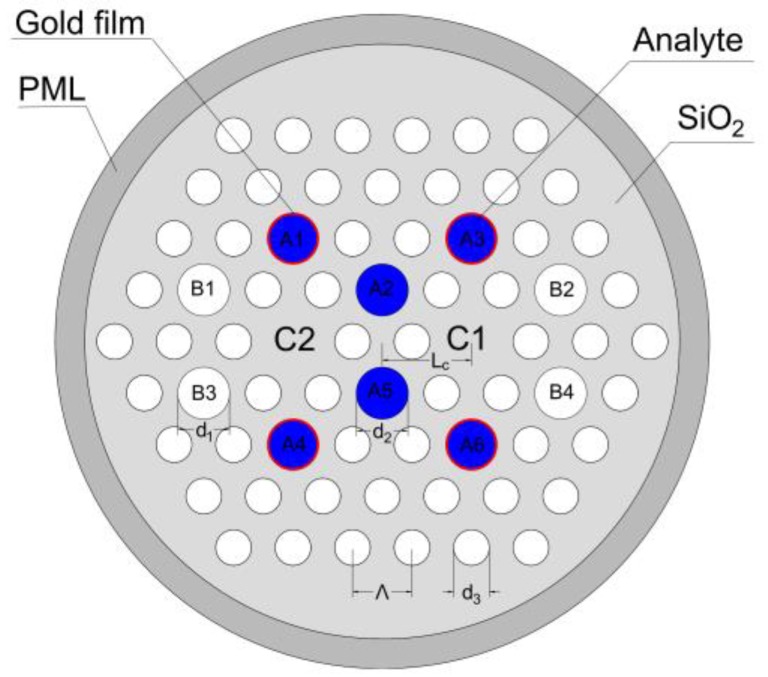
Schematic of the sensor.

The schematic of the dual-core PCF sensor is shown as Figure 1. The air holes are arranged in hexagonal lattice with a space of Λ = 2 μm. The core C1 and C2 present symmetrical arrangement around the geometric center of the PCF and the distance (L_c_) from the center is 3 μm. The diameter of the B holes (B1–B4 in Figure 1) is d_1_, the diameter of the A holes (A1–A6 in Figure 1) is d_2_, and the diameter of other small air holes is d_3_ = 0.6 Λ = 1.2 μm. The B holes are coated selectively (see in Figure 1), and the thickness (t) of gold film is 40 nm. The background material is SiO_2_, and its RI is given by Sellmeier Equation [5]:

(1) $$n_{SiO_{2}}^{2}(\lambda )=1+\frac{A_{1}\lambda^{2}}{\lambda^{2}-B_{1}}+\frac{A_{2}\lambda^{2}}{\lambda^{2}-B_{2}}+\frac{A_{3}\lambda^{2}}{\lambda^{2}-B_{3}}$$

Here, *λ* is the wavelength of the incident electromagnetic wave, and *A*_1_ = 0.696166300, *A*_2_ = 0.407942600, *A*_3_ = 0.897479400, *B*_1_ = 4.67914826 × 10^−3^ μm^2^, *B*_2_ = 1.35120631 × 10^−2^ μm^2^, *B*_3_ = 97.9340025 μm^2^, respectively. The dielectric constant of gold is given by the Drude model [14], and the RI of analyte (*n*) varies from 1.33 to 1.51.

**Figure 2:**
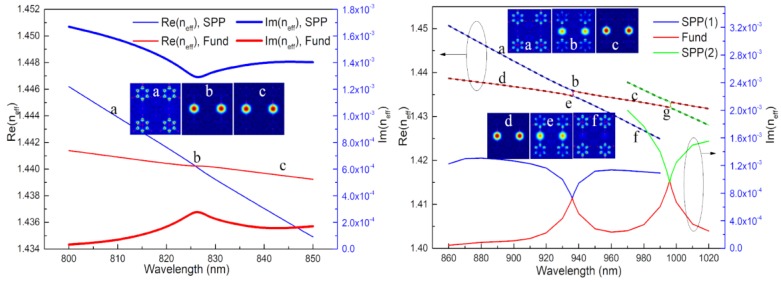
(**Left**): Dispersion relationship between the fundamental mode and the surface plasmon polaritons (SPP) mode with *n* = 1.46; (**Right**): Dispersion relationship between the fundamental mode and the SPP mode with *n* = 1.49.

The energy loss is caused by the appearance of surface plasmon resonance (SPR) between the gold film and the analyte. The absorption peaks change with the RI of an analyte. The analyte can be measured by judging the position of the absorption peak. The two adjacent core modes interact with each other, which is described as superimposition of electric fields, so they are called supermodes. The in-phase supermodes for which electric field distribution is in same direction are selected as the fundamental mode because their loss is less than that of other supermodes [17]. Figure 2 shows the dispersion relationships between the fundamental mode and the SPP mode at *n* = 1.46 (left) and *n* = 1.49 (right) when the A holes are filled with analyte and the B holes are empty, and the diameters of them are d_1_ = d_2_ = 2.6 μm. When the RI of an analyte is 1.46, the dispersion relationships intersect at point b (left graph, that is, the phase matching point), which results in incomplete coupling. In contrast, when the RI of the analyte is 1.49, the imaginary part curves of them are very close, and the real part difference is minimum at point e, which is called the avoided crossing point or the anti-crossing point. It can be considered that they undergo energy exchange intensively at point e and the anti-crossing effect takes place; this is known as complete coupling. It should be noted that the curves are depicted in the same color in the right illustration of Figure 2 in order to facilitate analysis. Indeed, the real part curves do not intersect as the dashed line shows in this picture. There are many resonance peaks; that is, there is more than one phase matching point, which was analyzed in ref. [1]. In the case of the complete coupling, there are also multiple peaks, as shown in Figure 2(right) where there are two avoided crossing points, e and g, in the range of 860 nm to 1020 nm. In this paper, only point e was selected as the avoided crossing point.

## 3. Results and Discussion

Variation in RI (*n*) led to the shift of the resonant peak. Loss is defined, according to ref. [5], as

(2) αloss(dB/m)=40πIm[neff]ln(10)λ,

where *λ* is the wavelength of the incident electromagnetic wave and *n_eff_* is the effective RI of the fundamental mode.

**Figure 3:**
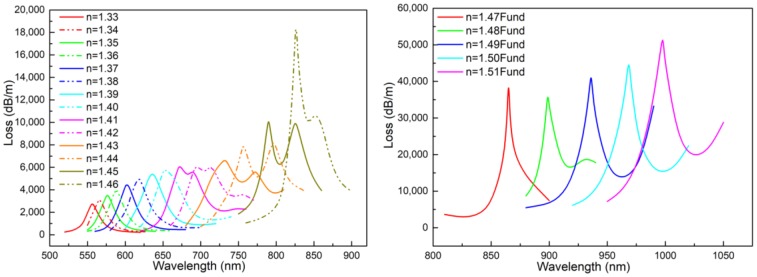
(**Left**): Loss when the refractory index (RI) of analyte (*n*) changes from 1.33 to 1.46; (**Right**): Loss when n varies from 1.47 to 1.51.

When the A holes were filled with analyte and the B holes were empty and both had diameters of 2.6 μm, and the change in loss curves with n varied from 1.33 to 1.46 and from 1.47 to 1.51, as shown as Figure 3(left),(right), which satisfies the phase matching condition and the loss matching condition, respectively. The absorption peak had a redshift with the increase in the RI of the analyte. That is, because the effective RI of the excited surface plasma mode increased, this led to a redshift of the phase matching point or the avoided crossing point. The sub-peak appeared near the loss peak when the RI was greater than 1.41, which made the loss curve more identifiable. Additionally, the redshift of the sub-peak can be used for auxiliary detection.

**Figure 4:**
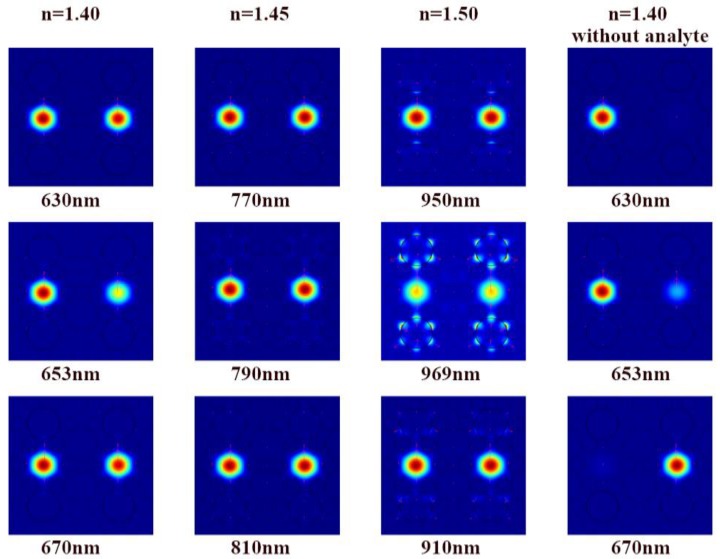
Fundamental mode profiles for different analyte RIs (the second line of the profiles is at the phase matching point or at the avoided crossing point except for the fourth profile. The two large holes near the center have no analyte in the fourth column.).

**Figure 5:**
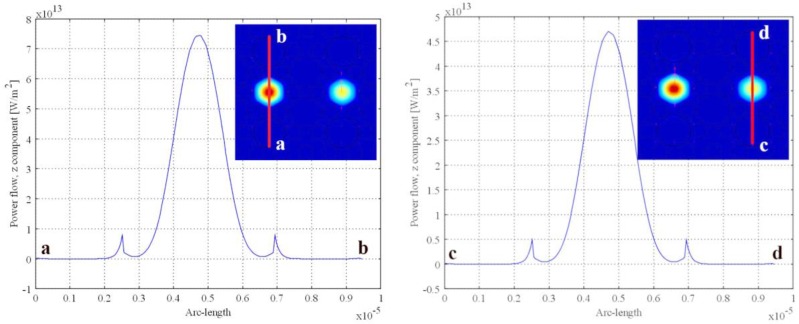
(**Left**): Power flow distribution along the red line from point a to point b with *n* = 1.40 at the phase matching point (a and b in the illustration are the head and terminal of the red line, respectively); (**Right**): Power flow distribution along the red line from point c to point d with *n* = 1.40 at the phase matching point. (c and d in the illustration are the head and terminal of the red line, respectively).

As is shown in the first column of Figure 4, the energy distribution of the two cores with *n* = 1.40 was not consistent around the phase matching point (653 nm), which shows that the energy in one core was greater than that in the other core. The coupling between the two adjacent fundamental modes led to a change in the complex amplitude of the light field in each core, so one of the two mutual coupling fundamental modes was disturbed by the other fundamental mode, especially when energy transferred from the fundamental mode to the SPP mode. Consequently, the two power flow distributions of the two cores were different, which is depicted as Figure 5. The disturbance was weakened either far away from the phase matching point (such as 630 nm and 670 nm in Figure 4) or at higher RIs (such as *n* = 1.45 and *n* = 1.50 in Figure 4), so that the energy distribution of the two cores was almost the same. As a comparison, the energy was confined by one core and the other core had little energy when A2 and A5 were not filled with analyte, as shown in the fourth column in Figure 4. This means air holes weaken the mode coupling between the two fundamental core modes, leading to the absence of supermodes. The loss in complete coupling (*n* ≥ 1.47) was much greater than the loss in incomplete coupling. For example, the fundamental mode energy was converted into the plasma mode with *n* = 1.50 at the avoided crossing point (*λ*_res_ = 969 nm) in the third column of Figure 4, which resulted in a significant energy decrease in the core and thus, the loss increased.

**Figure 6:**
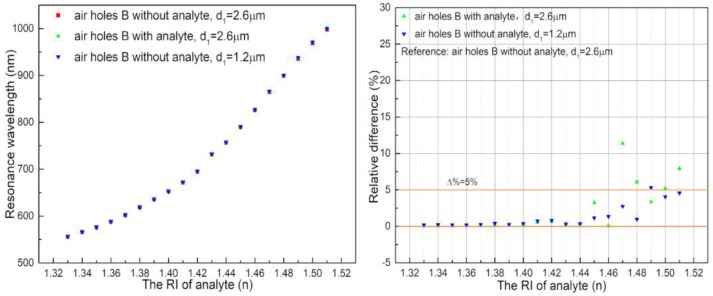
(**Left**): Scatter plot of the RI wavelength with B holes under different conditions; (**Right**): relative difference of the loss peak with B holes under different conditions.

The effects of filling the analyte and having different d_1_ values on the sensor are discussed. As shown in Figure 6, the B holes were not filled with analyte and the d_1_ was 2.6 μm, which was used as a reference to compare with other cases. For cases where the d_1_ was fixed at 2.6 μm, the scatter plots of the RI wavelength for B holes were filled with analyte (green) and not filled with analyte (reference, red) are coincident in the left of Figure 6. Similarly, under the condition where the B holes were not filled with analyte, the scatter plots of the RI wavelength with d_1_ = 1.2 μm (blue) and d_1_ = 2.6 μm (reference, red) were almost coincident as well. So, the impact of the B holes on the resonant wavelength is negligible; that is, the holes far away from the geometry center of the PCF have an insignificant influence on the wavelength sensitivity.

Here, the relative difference of the loss peak is defined as

(3) $$\Delta \%=\frac{\left|\alpha_{loss1}-\alpha_{loss2}\right|}{\alpha_{loss1}}\times 100\%$$

where, *α_loss_*_1_ is the loss peak of the reference when the B holes are not filled with analyte, and the d_1_ is 2.6 μm.

The right diagram in Figure 6, shows the condition where the B holes were filled with analyte and the d_1_ was 2.6 μm (green). The relative loss difference value was less than 5% in the case of the incomplete coupling (*n* ≤ 1.46), and the loss with analyte was greater than without analyte in the simulated B holes. Similarly, the relative difference in the loss peak was larger in the complete coupling than in the incomplete coupling when the B holes did not contain analyte and the d_1_ was 1.2 μm (blue). Therefore, the B holes have an effect on the loss peak only to a small extent in complete coupling, and their effect on the resonant wavelength is negligible. From the viewpoint of fiber fabrication, it is clear that the diameter of the B holes is 1.2 μm, in which case the B holes are the same as other small air holes of cladding. The following analysis is based on the condition where the B holes did not contain analyte and the d_1_ was 1.2 μm.

The A holes, as in liquid holes, have a great influence on the sensitivity. Using wavelength interrogation, the wavelength sensitivity can be calculated in accordance with ref. [5]:

(4) $$S_{\lambda }(nm/RIU)=\partial \lambda_{\mathrm{res}}/\partial n$$

where, *λ*_res_ is the resonance wavelength.

The detectable index resolution of the sensor is defined according to ref. [5]:

(5) $$R(RIU)=\Delta \lambda_{\min}\Delta n/\Delta \lambda_{res}=\Delta \lambda_{\min}/S_{\lambda }$$

where, Δ*λ*_min_ is the spectral minimum resolution.

**Figure 7:**
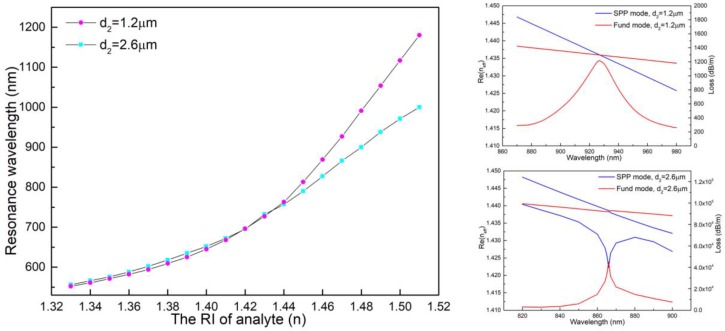
(**Left**): Resonance wavelength with variation in *n*. (**Right**): Dispersion relationship of the fundamental mode and the SPP mode at d_2_ = 1.2 μm and d_2_ = 2.6 μm with *n* = 1.47.

The RI wavelength curves for when the diameter of the A holes (d_2_) was 2.6 μm and 1.2 μm, respectively, are shown in Figure 7. The slope of the curve at d_2_ = 2.6 μm was smaller than that at d_2_ = 1.2 μm—the larger the d_2_, the smaller the sensitivity. The illustrations describe the dispersion relationships between the fundamental mode and the SPP mode at *n* = 1.47. The resonance peak occurred in the avoided crossing point at d_2_ = 2.6 μm, while the peak appeared in the phase matching point at d_2_ = 1.2 μm. In fact, the resonance peak at d_2_ = 1.2 μm appeared merely in the phase matching point with the RI of analyte increasing from 1.33 to 1.51. That means that the strength of coupling between the fundamental mode and the SPP mode of the sensor with small liquid holes was much less than that with larger liquid holes. Supercontinuum broadband source (SBS) was used as the light source because the designed sensor works at visible and near infrared wavelengths. As shown in Figure 7, however, the resonance wavelength varied nonlinearly when the RI of analyte was less than 1.44. It is time-consuming and inconvenient to recalculate the relationship between the resonant wavelength and the refractive index of the analyte in a specific refractive index range. Generally, the transfer function of the sensor with linear variation is more easily described than the nonlinear variation. The RI-wavelength curve with high linearity was in the range of 1.44 to 1.51.

**Figure 8:**
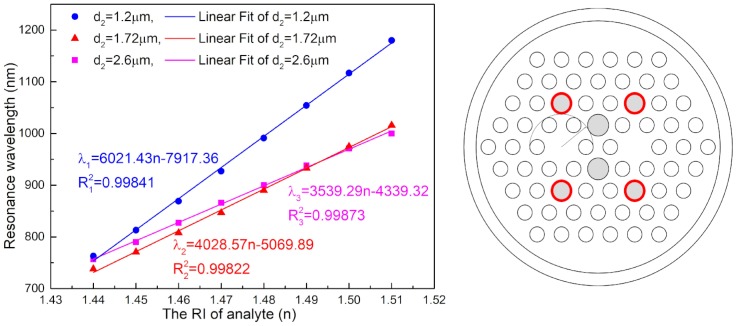
(**Left**): Resonance wavelength with *n* varying from 1.44 to 1.51; (**Right**): Structure of the improved sensor.

Several types of sensors that have been investigated in recent years are listed in Table 1. The comparison shows that the designed sensor has the advantage of higher RI detection. A resonance wavelength with the n varying from 1.44 to 1.51 is drawn in Figure 8. When the diameter of the liquid holes (d_2_) was 1.2 μm, the A holes were the same as other small air holes. The sensitivity was 6021 nm/RIU and the R^2^ was 0.99841. When the d_2_ was 2.6 μm, the A holes were nearly tangential with the other cladding air holes. The sensitivity was 3539 nm/RIU and the R^2^ was 0.99873. The linearity of the two was good, and the sensitivity of the former was higher than that of the latter. However, the smaller diameter made filling with analyte difficult. Taking into account the reduced filling difficulty and improving the sensitivity of the sensor, the value of d_2_ was determined to be between 1.2 μm and 2.6 μm. As shown in the right of Figure 8, the d_2_ was 1.72 μm; that is, the A holes were tangential with the envelope of the first layer cladding air hole. The diameters of d_1_ and d_3_ were both 1.2 μm in this setting. The d_2_ is increased by 43.33% compared to the smallest liquid holes. The wavelength sensitivity was as high as 4028 nm/RIU and the R^2^ was 0.99822, with n changing from 1.44 to 1.51, which shows that the sensor has high sensitivity and good linearity. If the spectral minimum resolution (Δ*λ*_min_) is 0.1 nm, the resolution of the sensor is 2.48 × 10^−5^ RIU. This structure not only makes it easier to fill with analyte, but it also has high sensitivity.

## 4. Conclusions

The influence of the parameters of the dual-core PCF was numerically simulated, and an optimized structure was proposed. The dynamic detection range of the sensor was from 1.33 to 1.51, in which the optimum detection range was shown to be from 1.44 to 1.51 because of the high sensitivity and good linearity in this range. The holes far away from the geometry center of the PCF had little impact on the performance of the sensor, and the diameter of the liquid holes affected the sensitivity of the sensor—the smaller the diameter of the liquid holes, the higher the sensitivity, and the maximum sensitivity was 6021 nm/RIU. It is convenient to fill analyte by adjusting the diameter of liquid holes so that they are tangential to the envelope of the first layer cladding holes. In this case, the sensitivity was 4028 nm/RIU and the R^2^ was 0.99822 when the n varied from 1.44 to 1.51, which means it has both high sensitivity and good linearity.

## Figures and Tables

**Table 1:** Comparison of the proposed sensor with existing sensors.

Sensor	Refractive Index Range	Wavelength Sensitivity (nm/RIU)	R^2^	Structure of the Photonic Crystal Fiber (PCF)
Ref. [3]	1.33–1.42	16,400 (maximum)	(Nonlinearity)	Exposed-core grapefruit PCF
Ref. [8]	1.33–1.37	5200	(Not given)	D-shaped PCF
Ref. [12]	1.39–1.42 1.43–1.46	4350 9200	0.99698 0.99864	Dual-core PCF
Ref. [14]	1.34–1.37	4400	0.9584	Circular lattice PCF
Ref. [30]	1.36–1.41	14,660 (average)	(Not given)	Dual D-shaped PCF
This work (d_2_ = 1.2 μm)	1.44–1.51	6021	0.99841	Dual-core PCF
This work (d_2_ = 1.72 μm)	1.44–1.51	4028	0.99822	Dual-core PCF
